# Status and Influencing Factors of Parenteral Nutrition Practice for Late Preterm Infants in China

**DOI:** 10.3389/fped.2022.844460

**Published:** 2022-03-08

**Authors:** Meiying Quan, Zhenghong Li, Danhua Wang, Kurt Schibler, Li Yang, Jie Liu, Xuanguang Qin, Xin Zhang, Tongyan Han, Ying Li

**Affiliations:** ^1^Peking Union Medical College Hospital, Department of Pediatrics, Beijing, China; ^2^Cincinnati Children’s Hospital Medical Center, Cincinnati, OH, United States; ^3^Department of Pediatrics, Beijing Tongzhou District Maternal and Child Health Hospital, Beijing, China; ^4^Department of Pediatrics, The People’s Hospital of Beijing University, Beijing, China; ^5^Department of Pediatrics, Beijing Chaoyang Hospital, Beijing, China; ^6^Department of Pediatrics, Peking University First Hospital, Beijing, China; ^7^Department of Pediatrics, Peking University Third Hospital, Beijing, China; ^8^Department of Pediatrics, Beijing Haidian District Maternal and Child Health Hospital, Beijing, China

**Keywords:** late preterm infants, parenteral nutrition, influencing factors, growth, amino acid, lipid emulsion

## Abstract

**Objectives:**

To explore the status of parental nutrition practice of hospitalized late preterm infants and the factors influencing the clinical prescription.

**Methods:**

A multi-center, prospective cohort study was conducted during October 2015 to October 2017. Infants born after 34 weeks and before 37 weeks were enrolled from twenty-five hospitals in the Beijing area of China. Data of enteral and parenteral nutrition were collected.

**Results:**

A total of 1,463 late preterm infants were enrolled in this study, 53.9% of infants were supported by parenteral nutrition. Over 60% of 34 weeks’ infants were on parenteral nutrition during the 2nd to the 4th day. Logistic regression analysis showed that gestational age(GA) (OR = 0.69, 95%CI 0.58–0.81), birth weight (OR = 0.41, 95%CI 0.26–0.65), hypoglycemia (OR = 2.77, 95%CI 1.90–4.04), small for gestational age (SGA) (OR = 2.18, 95%CI 1.34–3.55), feeding intolerance (OR = 6.41, 95%CI 1.90–21.59), neonatal respiratory distress syndrome (NRDS) (OR = 2.16, 95%CI 1.12–4.18), neonatal infection(OR = 1.56 95%CI 1.16–2.10), and slow enteral nutrition advancement rate (OR = 0.92, 95%CI 0.90–0.95) were factors influencing the administration of parenteral nutrition.

**Conclusion:**

Over half of hospitalized late preterm infants were prescribed with parenteral nutrition. Infants with lower GA, lower birth weight, diagnosed with hypoglycemia, SGA, feeding intolerance, NRDS, neonatal infection, or a slower rate of enteral nutrition advancement had a higher likelihood of receiving parenteral nutrition.

## Introduction

Nutritional practices in early life may impact short-term complications of preterm infants as well as their later neurologic and metabolic health; therefore, nutritional support is an important part of comprehensive management for preterm infants (1–3). Nutritional support of extremely and very low birth weight preterm infants (infants with birth weight <1000g and <1500g, respectively) has been studied extensively, leading to consensus and guidelines on the optimal enteral and parenteral nutrition strategies (4, 5). Late preterm infants are the largest population among preterm infants, who may cost the most medical resources, but conditions could not be compared to those extremely immature infants. There is little high-quality evidence based nutritional recommendation on these infants (2, 6). Nutritional practices vary widely throughout the world, even in the same medical institution (7–9). For instance, while waiting for full enteral feeds to be tolerated, there are no data on whether these late preterm infants should receive parenteral nutrition, like that used for extremely preterm infants.

We conducted a multi-center clinical study to explore the status of nutritional practices of hospitalized late preterm infants in the Beijing area of China. For enteral nutrition, previous data showed that exclusive human milk feeding rate was as low as 4.5% and about half of late preterm infants did not regain birth weight at discharge (10). Parenteral nutrition is an indispensable nutritional support for certain late preterm infants, the parenteral nutrition strategy and their impact factors will be explored here.

## Materials and Methods

### Subjects

Preterm infants born at 34^+0^ through 36^+6^ weeks gestational age (GA) between October 2015 to October 2017 and admitted to the neonatal intensive care units (NICUs) or neonatal nurseries of 25 hospitals within the first 3 days of life were included.

Exclusion criteria included infants with major congenital anomalies and/or gastrointestinal diseases, infants who transferred to other medical institutions before discharge or died within the first 72 h of life.

### Study Design and Definitions

This is a multi-center study conducted based on data from 25 member hospitals affiliated to Beijing Cooperative Multi-Center Preterm Infants Network. The 25 member hospitals include 20 level III hospitals and 5 level II hospitals. Written informed consent was obtained by parents at time of infants’ admission. This trial was registered at www.clinicaltrials.gov as NCT02605577 on November 16, 2015.

The data collection form was designed as a software program installed in tablet computers and provided to all the participating hospitals. The length and head circumference measurement were standardized, and the weighing scales were calibrated. Standardized operation manuals were handout to participating hospitals containing instructions and the definitions. Complications are defined from “Practice of Neonatology (4th edition)” (11). Exclusive human milk feeding is defined as human milk feeding since birth, without supplementing any other formula. Human milk was calculated as 67 kcal/100 ml. Enteral nutrition included exclusive human milk feeding, formula feeding and mixed feeding. Infants receiving parenteral nutrition (including amino acids or lipid emulsion) were classified as PN-group. Infants with merely enteral feeds and/or carbohydrates infusion were classified as non-PN group.

### Data Collection and Analysis

Baseline data were obtained soon after admission, and outcome data were sent to database through everyday submission or at the time the infants were discharged. Neonatologists in charge of the enrolled infants completed the medical charts after completing daily medical visit. Basic data included GA (based on the last menstrual period and first-trimester ultrasonogram), gender, Apgar score, birth weight (BW), length, head circumference, whether diagnosed as small for gestational age (SGA), asphyxia, hypothermia, neonatal respiratory distress syndrome (NRDS), apnea, transient tachypnea of newborn, hyperbilirubinemia, hypoalbuminemia, anemia, neonatal infection, early and late-onset sepsis, meningitis, hypoglycemia, hyperglycemia, feeding intolerance, necrotizing enterocolitis (NEC) and intra-ventricular hemorrhage (IVH) during hospital stay. These complications were diagnoses or emerged before the application of parenteral nutrition.

The time regaining birth weight, weight at discharge, length at discharge, head circumference at discharge, and weight growth velocity (GV) were recorded and calculated, as well as the average enteral nutrition advancement rate.

$G\,V=1000\times \ln\left(\frac{w\,e\,i\,g\,h\,t\,y}{w\,e\,i\,g\,h\,t\,x}\right)/\left(D\,y-D\,x\right)$, *x* = the day with the lowest body weight, *y* = length of stay (12, 13).

Enteral nutrition advancement rate = (enteral feeding volume at discharge −initial feeding volume)/(enteral feeding duration−1).

### Statistics

The Statistical Package for the Social Sciences (SPSS) for Windows, version 24.0 (IBM SPSS Inc., Chicago, IL, United States, RRID: SCR_019096) was used for statistical analysis. The Kolmogorov–Smirnov test was used to determine whether the variables were normally distributed. Data were presented as mean ± standard deviation for normally distributed variables, and percentages (median, and P_25_, P_75_) for variables that were not normally distributed. Comparisons of categorical variable between two or more groups were performed by the χ^2^ test or fisher exact test. Comparisons of continuous variables that was not normally distributed between two groups were performed by Mann–Whitney test. Comparisons of continuous variables that among three groups were performed by ANOVA for normal distribution data or Kruskal–Wallis test for abnormal distribution data. Binary logistic regression was also used to determine the influencing factors of parenteral nutrition. The level of significance was set at *p* < 0.05.

## Results

### Demographic Data of Late Preterm Infants

From October 2015 to October 2017, a total of 1,463 late preterm infants were enrolled in this study, with a median GA of 35.6 (34.9, 36.1) weeks. Basic characteristics are shown in Table 1. Incidence of SGA, Apnea and NRDS had statistically significant differences between infants of 34, 35, and 36 weeks of GA, with higher incidence of apnea and NRDS in 34 weeks infants. Incidence of SGA was higher in 36 weeks group. Hospital-stay was longer among infants of 34 weeks of GA.

**TABLE 1:** Demographic data of late preterm infants.

	Enrolled infants (*n* = 1463)	34 wks (*n* = 369)	35 wks (*n* = 566)	36 wks (*n* = 528)	Statistics	*P*-value
N (%)	1463(100%)^1^	369(25.2%)	566(38.7%)	528(36.1%)		
GA (weeks)	35.6(34.9,36.1)^2^	34.4(34.1,34.7)	35.4(35.1,35.7)	36.3(36.1,36.6)		
Male infants n (%)	789(53.9%)	207(56.1%)	303(53.5%)	279(52.8%)	2.668	0.611
C-section n (%)	962(65.8%)	262(71.0%)	351(62.0%)	349(66.1%)	9.062	0.047
SGA (%)	170(11.6%)	28(7.6%)	52(9.2%)	90(17.0%)	22.439	<0.001
Resuscitation in DR	79(5.4%)	23(6.2%)	23(4.1%)	33(6.2%)	7.400	0.216
Birth weight(g)	2440(2200,2710)	2250(2020, 2440)	2485(2259, 2740)	2545(2300, 2800)	154.087	<0.001
Birth length (cm)	47.0(45.0,48.0)	46.0(44.0, 47.0)	47.0(45.0, 48.0)	47.0(46.0, 49.0)	92.134	<0.001
Birth HC (cm)	32.0(31.5,33.0)	32.0(30.5, 33.0)	32.5(32.0, 33.0)	32.5(32.0, 33.0)	90.266	<0.001
Hypothermia (%)	8(0.6%)	1(0.3%)	4(0.7%)	3(0.6%)	0.727	0.695
Neonatal infection	324(22.2%)	85(23.1%)	127(22.4%)	112(21.3%)	0.432	0.806
Early-onset sepsis	12(0.8%)	4(1.2%)	1(0.2%)	7(1.3%)	4.370	0.112
Late-onset sepsis	4(0.3%)	2(0.6%)	0(0.0%)	2(0.4%)	2.846	0.241
Apnea	38(2.6%)	20(5.3%)	12(2.1%)	7(1.3%)	14.006	0.001
TTN	124(8.5%)	39(10.7%)	44(7.8%)	40(7.6%)	2.896	0.235
NRDS	60(4.1%)	26(7.1%)	17(3.0%)	17(3.2%)	10.640	0.014
Feeding intolerance	50(3.4%)	15(4.2%)	18(3.2%)	17(3.2%)	0.750	0.687
NEC	5(0.4%)	1(0.3%)	2(0.4%)	2(0.4%)	0.083	0.960
IVH	74(5.1%)	25(6.8%)	28(5%)	21(4%)	3.251	0.197
Length of hospital stay(d)	8(7,11)	10(8,13)	8(7,10)	7(6,9)	163.184	<0.001

*GA, gestational age; HC, head circumference; SGA, small for gestational age; NEC, necrotizing enterocolitis; NRDS, neonatal respiratory distress syndrome; IVH, intraventricular hemorrhage; TTN, transient tachypnea of neonate; Infection, infants that needs antibiotics therapy more than 3 days. ^1^Number(percentage) (all such values) and χ^2^ test or fisher was used for comparison between groups. ^2^Median; 25th–75th percentile in parentheses (all such values) and Kruskal–Walli test was used for comparison between groups.*

### Features of Parenteral Nutrition

**FIGURE 1:**
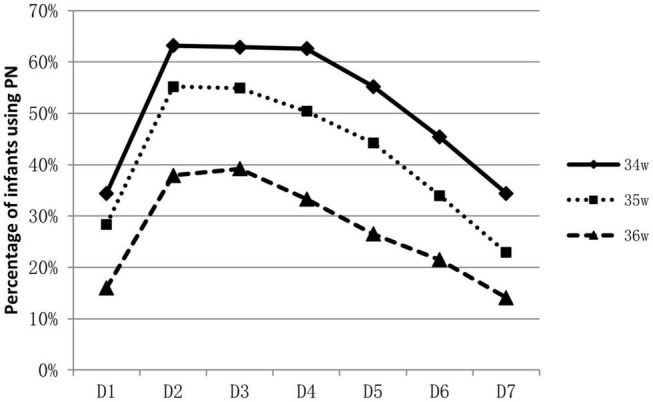
Percentage of late preterm infants who were applied with PN during hospitalization and the changes over time.

**FIGURE 2:**
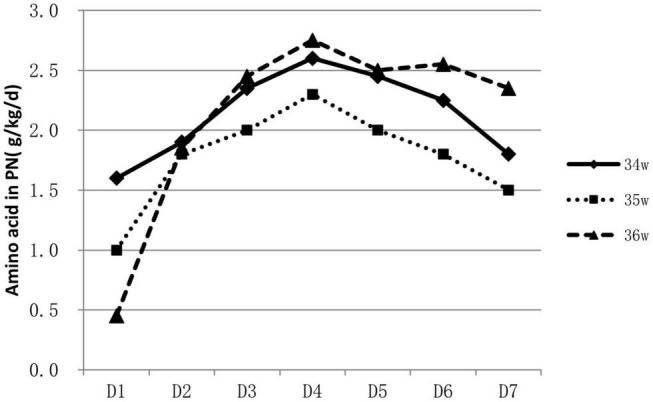
Characteristics of amino acid prescription in PN for hospitalized late preterm infants.

**FIGURE 3:**
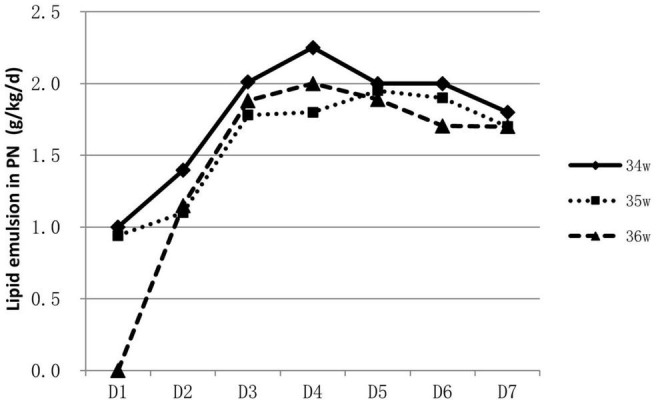
Characteristics of lipid emulsion prescription in PN for hospitalized late preterm infants.

About 53.9% (788/1463) of infants were supported by parenteral nutrition containing amino acids and lipid emulsion for more than one day. The proportion of infants on parenteral nutrition changed over time and was inversely related to GA. Over 60% of 34 weeks’ infants were on parenteral nutrition during the 2nd to the 4th day, At the end of the 1st week, infants on parenteral nutrition decreased to 10–40% (Figure 1). Infants on parenteral nutrition were prescribed with amino acid at 1.5–2.5 g/kg/d and lipid emulsion at 1.5–2.0 g/kg/d. The starting volume of amino acid and lipid emulsion in parenteral nutrition were related to GA, infants with lower GA tended to have higher volume of amino acids and lipid emulsion in parenteral nutrition (Figures 2, 3).

Univariate-analysis showed that GA, birth weight, birth length, birth circumference, incidence of SGA, feeding intolerance, hypoglycemia, NRDS, neonatal infection, apnea, anemia, growth velocity, volume of enteral nutrition at discharge and enteral nutrition advancing rate had significant difference between the parenteral nutrition (PN) and non-parenteral nutrition (non-PN) group (Table 2). Late preterm infants in PN group had lower enteral feeding volume from D1 to D7 as well as at the time of discharge but had higher exclusive human milk feeding rate.

**TABLE 2:** Risk factors of PN and influence of PN on late preterm infants during hospitalization.

	PN group (*n* = 788)	Non-PN group (*n* = 675)	Statistics	*P*-value
Male (%)	414(52.5%)^1^	379(56.1%)	3.12	0.210
GA (w)	35.2(34.6,35.9)^2^	35.7(34.9,36.2)	170931.0	<0.0001
Birth weight(g)	2287(2020,2500)	2435(2240,2732)	170338.5	<0.0001
Birth length (cm)	46(44,48)	47(45,48)	182454.5	<0.0001
Birth HC (cm)	32(31,33)	32(31.5,33)	185926.5	<0.0001
SGA (%)	123(15.6%)	38(5.6%)	32.323	<0.0001
Asphyxia (%)	13(1.6%)	8(1.2%)	0.419	0.517
Feeding intolerance (%)	45(5.7%)	3(0.4%)	27.367	<0.0001
Hypoglycemia (%)	140(17.8%)	59(8.7%)	22.88	<0.0001
NEC (%)	3(0.4%)	2(0.3%)	0.035	0.851
NRDS (%)	43(5.5%)	16(2.4%)	8.14	0.004
Transient tachypnea of newborn (%)	66(8.4%)	58(8.6%)	0.017	0.897
Infection (%)	202(25.6%)	121(17.9%)	11.357	0.001
Early onset sepsis (%)	9(1.2%)	2(0.3%)	3.036	0.081
Late onset sepsis (%)	3(0.4%)	1(0.1%)	0.597	0.440
Apnea (%)	29(3.7%)	8(1.2%)	8.509	0.004
Hypoalbunemia (%)	19(2.4%)	24(3.6%)	1.527	0.217
Anemia (%)	139(17.6%)	74(11.0%)	11.592	0.001
Hypothermia (%)	6(0.8%)	2(0.3%)	1.199	0.274
IVH (%)	45(5.7%)	30(4.4%)	1.239	0.266
Growth velocity(g/kg/d)	11.2(7.6,15.1)	8.5(4.8,12.5)	135220.5	<0.0001
Volume of EN on D1 (ml/kg/d)	10.3(5.1,15.8)	19.2(6.7,32.0)	126471.0	<0.0001
Volume of EN on D2 (ml/kg/d)	19.7(12.6,32.4)	41.4(27.2,56.7)	95768.5	<0.0001
Volume of EN on D3 (ml/kg/d)	30.5(17.3,46.0)	60.6(41.5,80.3)	93083.0	<0.0001
Volume of EN on D4 (ml/kg/d)	47.9(28.9,65.6)	82.5(59.3,106.2)	83907.5	<0.0001
Volume of EN on D5 (ml/kg/d)	63.4(39.8,85.1)	102.4(79.1,121.2)	76543.0	<0.0001
Volume of EN on D6 (ml/kg/d)	78.4(54.5,102.9)	119.6(96.8,138.4)	63248.5	<0.0001
Volume of EN on D7 (ml/kg/d)	88.6(64.1,114.9)	129.5(107.5,155.1)	50744.5	<0.0001
Volume of EN at discharge (ml/kg/d)	128.6(98.9,157.0)	144.7(124.8,170.1)	195205.5	<0.0001
EN advancing rate (ml/kg/d)	11.3(8.8,13.9)	14.6(12.2,17.9)	151706.5	<0.0001
Exclusive HM feeding rate (%)	41(5.2%)	25(3.7%)	1.682	0.195
Infants regain BW at discharge (%)	553(70.2%)	322(47.7%)	69.741	<0.0001
Length of hospital stay (d)	9(7,12)	8(6,9)	135396.0	<0.0001

*GA, gestational age; HC, head circumference; SGA, small for gestational age; NEC, necrotizing enterocolitis; NRDS, neonatal respiratory distress syndrome; IVH, intraventricular hemorrhage. ^1^Number(percentage) (all such values) and χ^2^ test or fisher was used for comparison between groups. ^2^Median; 25th–75th percentile in parentheses (all such values) and Mann–Whitney test was used for comparison between groups.*

Late preterm infants with PN had higher growth rate [11.2 (7.6,15.1) g/kg/d vs. 8.5 (4.8,12.5) g/kg/d, *p* < 0.0001] and higher possibility to regain birth weight at discharge than infants without PN (70.20% vs. 47.70%, *p* < 0.0001).

Binary logistic regression analysis showed that infants with lower GA or lower birth weight, infants diagnosed with hypoglycemia, SGA, feeding intolerance, NRDS, neonatal infection, and infants with lower enteral nutrition advancement rate had higher possibility to start parenteral nutrition (Table 3).

**TABLE 3:** Bilary logistic regression analysis of PN application for late preterm infants.

	B	S.E.	Wald	OR	95% *C.I*	*P*-value
GA(week)	−0.371	0.087	18.08	0.69	0.581–0.819	<0.0001
Birth weight(kg)	−0.886	0.235	14.235	0.412	0.26–0.653	<0.0001
Birth length(cm)	−0.001	0.028	0.001	0.999	0.946–1.055	0.97
Birth circumference(cm)	0.059	0.037	2.527	1.06	0.986–1.14	0.112
Feeding intolerance (Yes = 1)	1.858	0.62	8.988	6.409	1.903–21.586	0.003
Hypoglycemia (Yes = 1)	1.02	0.192	28.265	2.773	1.904–4.039	<0.0001
NRDS (Yes = 1)	0.77	0.337	5.234	2.16	1.117–4.178	0.022
Apnea (Yes = 1)	0.562	0.46	1.491	1.754	0.712–4.322	0.222
Neonatal infection (Yes = 1)	0.444	0.153	8.447	1.559	1.156–2.104	0.004
Anemia (Yes = 1)	0.333	0.183	3.323	1.395	0.975–1.996	0.068
SGA (Yes = 1)	0.779	0.25	9.752	2.18	1.337–3.554	0.002
Enteral nutrition advancing rate(ml/kg/d)	−0.077	0.012	43.442	0.926	0.905−0.947	<0.0001

*Dependent variable: PN application = 1, non-PN = 0; GA, gestational age; NRDS, neonatal respiratory distress syndrome; SGA, small for gestational age.*

## Discussion

### Status of Parenteral Nutrition Support on Late Preterm Infants in China

Enteral nutrition, especially breast milk, is the first option for late preterm infants, but sometimes, it may not be established as expected. Except for the separation of mother and sick late preterm infant and the insufficient attention on benefit of exclusive human milk feeding, the coordinated sucking ability that allows for the provision of sufficient intake by sucking feeds is not well developed until 34 weeks (14). And that the hepatic glycogen stores double between 36 and 40 weeks, this also places late preterm infants at risk of hypoglycemia in the postnatal period due to insufficient endogenous energy stores. Data showed that feeding problems were the primary reason for prolonged hospital stay, together with critical illness status, up to 75% of late preterm infants required additional feeding support, which may be gastric tube feeding, carbohydrates infusion or even parenteral nutrition (15). However, providing only carbohydrates in the first few days following birth to whom enteral nutrition is not established or not adequate, will likely lead to protein deficiency and a nitrogen deficit as obligatory protein demand equates to at least 0.7 g/kg/day (16). The malnutrition of preterm infants during early life would influence the short-term and long-term prognosis. Accordingly, when enteral nutrition is contraindicated or predicted to have nutrient insufficiency, parenteral nutrition is usually considered due to the essential amino acid, lipids, carbohydrates and energy it could provide.

For extremely preterm infants, the parenteral nutrition is initiated as soon as possible after delivery to prevent nutrition deficit (16, 17), while for late preterm infants, the indication, start time and component of parenteral nutrition varies greatly (18). In a study conducted in Italy, of the total of 1,768 late preterm infants included, 592 infants required nutritional support, including infants that had needed either parenteral nutrition or intravenous fluids (19). A survey conducted among caregivers of late preterm infants in New Zealand showed that 33% (25/76) commenced parenteral nutrition within 24 h of admission, 27% (20/75) between 24 and 48 h, 24% (18/75) between 48 and 72 h, 9% (7/75) between 72 and 96 h, and 4% (3/75) between 96 h and 7 days. For the prescription of parenteral nutrition, 61% (46/75) aimed for 1.5–3 g/kg/d of amino acids, whereas 27% (20/75) aimed for 2–3 g/kg/d. Eighty-three percent (63/76) aimed for a dose of 2.5g–3.5 g/kg/d of lipids; about 9 % (7/76) targeted a dose of 1–2.5 g/kg/d and 4% (3/76) for >3.5 g/kg/d (20). Data in our study showed that over half of the late preterm infants were given parenteral nutrition support for at least one day. Most concentrated on the second day to the fourth day after birth. The need for parenteral nutrition was inversely related to GA, over 60% of 34 weeks’ infants were on parenteral nutrition during the second to the fourth day of life, while only 30–40% for infants of 36 weeks. At the end of the 1st week, infants on parenteral nutrition decreased to 10–40%. The composition of parenteral nutrition prescribed varied from intravenous infusion of carbohydrates alone to combinations of dextrose, lipids, and amino acids in addition to minerals and vitamins (16, 20). Infants were prescribed with amino acid at 1.5–2.5 g/kg/d and lipid emulsion at 1.5–2.0 g/kg/d at the end of the 1st week. Data from abroad and China mostly depicted the status and variations of parenteral nutrition application, and lack of consensus and guidelines. More high-quality studies are needed to illustrate what are the optimal indication and protocol for the clinical application of parenteral nutrition on late preterm infants, and the influence of the strategy on their long-term prognosis.

### Factors Influencing Clinical Decision of Parenteral Nutrition and Its Impact on Growth

Many factors influenced the clinical decision to initiate and sustain parenteral nutrition. Logistic regression analysis revealed that GA, birth weight, hypoglycemia, SGA, feeding intolerance, NRDS, neonatal infection, and low enteral nutrition advancement rate were independently associated with the application of parenteral nutrition.

Late preterm infants with lower GA and birth weight had higher possibility of immature gastrointestinal tract and medical deliberated slower enteral nutrition progress due to the worry of feeding intolerance. Parenteral nutrition became ineluctable sometimes, but lasted shorter duration than extremely premature infants. The starting volume of amino acid and lipid emulsion were also found to be related to GA; infants with smaller GA tend to have higher volume of amino acid and lipid emulsion infusion as shown in our study.

The occurrence of medical problems in infants born with a GA of 34–36 weeks was significantly higher than in infants born at term. Accordingly, another indication of parenteral nutrition were clinical comorbidities such as hypoglycemia, SGA, feeding intolerance, NRDS, and neonatal infection like that revealed in our study. These findings were in agreement with previous data reported in literature from Italy, which showed that birth weight ≤2000 g, GA of 34 weeks and being born SGA, infants having developed a respiratory distress syndrome and having required a surgical intervention resulted to be independently associated with a higher risk of receiving a nutritional support. In some institution, protocols were set as infants with a birth weight <1500 g and infants presenting with any clinical condition that could hinder the beginning of enteral nutrition or that could interfere with the ability to feed exclusively by mouth (19).

The immaturity status, birth weight, clinical comorbidities and the concern of feeding problem are main factors that may influence the decisions of initiating parenteral nutrition. Nevertheless, the aim of additional parenteral nutrition application was to improve growth of late preterm infants and avoid the side effect of malnutrition. Our study demonstrated that late preterm infants with enteral nutrition plus parenteral nutrition had higher growth rate (11.2(7.6,15.1) g/kg/d vs. 8.5(4.8,12.5) g/kg/d, *p* < 0.0001) and higher possibility to regain birth weight at discharge (70.20% vs. 47.70%, *p* < 0.0001). However, the causes of initiating parenteral nutrition did not negatively affect the growth of these late preterm infants. When enteral nutrition advancement is not as good as expected, parenteral nutrition should be initiated to ensure the total energy intake and optimal growth (2, 21–23). But the complications associated with long term parenteral nutrition, such as cholestasis and catheter related infection, should be taken into account and regular assessment should be performed.

### Limitations

This multi-center prospective cohort study lacks long term follow-up data of these late preterm infants, especially in terms of neurodevelopmental and metabolic prognosis. Optimal nutritional strategy for late preterm infants to achieve better neurodevelopment and body composition should be investigated in well-designed randomized controlled trials.

## Conclusion

Over half of hospitalized late preterm infants were supported by parenteral nutrition. The application of parenteral nutrition concentrated on the second to the fourth day. Infants with lower GA or lower birth weight, and infants diagnosed with hypoglycemia, SGA, feeding intolerance, NRDS, neonatal infection, slower enteral nutrition advancing rate had higher possibility to start parental nutrition. Late preterm infants with enteral nutrition plus parenteral nutrition had higher growth rate than infants with exclusively enteral feeding support.

## Data Availability Statement

The raw data supporting the conclusions of this article will be made available by the authors, without undue reservation.

## Ethics Statement

The studies involving human participants were reviewed and approved by Ethical Committee of Peking Union Medical College Hospital. Written informed consent to participate in this study was provided by the participants’ legal guardian/next of kin.

## Author Contributions

MQ collected the data, performed the analysis, and drafted the initial version of the manuscript. ZL had primary responsibility for the study design, data analysis, and interpretation, and reviewed and revised the manuscript. DW and KS supervised the data collection and were involved in data interpretation. LY, JL, XQ, XZ, TH, and YL was involved in software and data collection. All authors contributed to the article and approved the submitted version.

## Conflict of Interest

The authors declare that the research was conducted in the absence of any commercial or financial relationships that could be construed as a potential conflict of interest.

## Publisher’s Note

All claims expressed in this article are solely those of the authors and do not necessarily represent those of their affiliated organizations, or those of the publisher, the editors and the reviewers. Any product that may be evaluated in this article, or claim that may be made by its manufacturer, is not guaranteed or endorsed by the publisher.
